# Evaluating the applicability of computational super-resolution techniques for harmonic generation microscopy

**DOI:** 10.1038/s41598-026-58983-0

**Published:** 2026-06-23

**Authors:** MacAulay Harvey, Richard Cisek, Sarry Al-Turk, Harry E. Ruda, Laurent Kreplak, Danielle Tokarz

**Affiliations:** 1 Department of Chemistry, Saint Mary’s University, 923 Robie Street, Halifax, NS B3H 3C3 Canada; 2 Department of Materials Science and Engineering, University of Toronto, 184 College Street, Toronto, ON M5S 3E4 Canada; 3 Department of Electrical and Computer Engineering, University of Toronto, 10 Kings College Road, Toronto, ON M5S 3G4 Canada; 4 Department of Physics and Atmospheric Science, School of Biomedical Engineering, Dalhousie University, Halifax, NS B3H 4J5 Canada

**Keywords:** Nanoscience and technology, Optics and photonics

## Abstract

Second harmonic generation and third harmonic generation (SHG and THG) microscopy are nonlinear optical imaging techniques which have found a diverse range of applications in the investigation of both biological and synthetic nanostructures. Like all optical imaging techniques, the spatial resolution achievable using SHG and THG is limited by diffraction to around half of the excitation wavelength, a major impediment towards applications in nano imaging. Because of this, several groups have developed methods for super-resolution SHG and THG imaging, however these approaches have all involved the use of nonstandard microscope components which presents a technical and financial barrier to entry for those interested in applying these techniques. Here we investigate the application of several computational super-resolution techniques for SHG and THG imaging, with a focus on enabling super-resolution polarization-resolved SHG microscopy. We find that even though computational super-resolution was originally developed for incoherent imaging modalities such as fluorescence, it is able to provide a lateral resolution enhancement of up to 3.4× when imaging isolated nanostructures compared to a standard laser scanning harmonic generation microscope. While currently available CSR techniques are not able, in general, to correct for the inherent ambiguity between density of emitters and signal intensity within coherent imaging processes, we show that CSR can still provide improved localization in harmonic generation microscopy. We additionally show that two computational super-resolution techniques, super-resolution radial fluctuations and deblurring by pixel reassignment, preserve the polarization dependence of SHG signal, thereby allowing super-resolution polarization-resolved SHG measurements. These results are obtained with no modification to our microscope system and little change to the experimental workflow, and therefore present exciting opportunities for future applications of super-resolution SHG and THG microscopy.

**Supplementary Information:**

The online version contains supplementary material available at https://doi.org/10.1038/s41598-026-58983-0.

## Introduction

The increasing availability of ultrafast pulsed laser sources over the past thirty years has led to the emergence of new microscopic imaging techniques utilizing the nonlinear optical processes of second harmonic generation (SHG), and third harmonic generation (THG). These techniques enable label-free characterization of biological and synthetic samples with microscopic spatial resolution, and the intrinsic optical sectioning granted by the nonlinearity of these processes can enable imaging deep within biological samples^1,2^. Like all optical imaging techniques, the lateral resolution which can be achieved in harmonic generation microscopy is limited by diffraction to around half of the wavelength of the excitation laser. This is a key disadvantage since SHG and THG have found major applications in the investigation of nano-scale samples, such as collagen fibrils, myofibrils and semiconductor nanowires for SHG^3,4^, and cellular organelles for THG^5^.

To circumvent this limitation a variety of approaches for super-resolution nonlinear optical imaging have been implemented including re-scan microscopy^6^, image scanning microscopy^7,8^, utilizing a photonic nanojet for SHG excitation^9^, multifocal structured illumination microscopy^10^ and selective deactivation of THG using a donut shaped beam^11^. While these hardware-based approaches have all enabled impressive resolution enhancements for SHG and THG imaging, they also require modifications to the microscope and the introduction of non-standard components in the excitation and/or collection pathways. This creates a major technical and financial barrier to entry for the application of these techniques, particularly for researchers interested in applications of nonlinear microscopy who lack specialized knowledge on microscope construction.

Computational super-resolution (CSR) techniques, which derive resolution enhancement from computational processing of microscopic images, present a possible avenue towards achieving super-resolution SHG/THG images without introducing any additional complexity to the imaging system. CSR approaches have been used extensively to enhance resolution in fluorescence microscopy, an incoherent imaging technique^12^. However thus far the application of these techniques for harmonic generation imaging, a coherent imaging technique, has been limited to deconvolution-based approaches^13,14^, which only offer limited resolution enhancement and often amplify noise. Recently several new CSR techniques have emerged which enhance resolution through analysis of intensity gradients within an image, and are able to provide greater resolution enhancement, and reduced noise compared to deconvolution. These techniques include super-resolution radial fluctuations (SRRF)^15^, mean-shift super-resolution (MSSR)^16^, and deblurring by pixel reassignment (DPR)^17^. While each of these techniques have found applications in super-resolved fluorescence imaging, they have not yet been applied to harmonic generation microscopy.

Here we investigate the applicability of these CSR techniques to harmonic generation microscopy, with a specific focus on enabling super-resolution polarization-resolved SHG (PSHG) imaging. First, we investigate the impact of interference between point sources on the CSR output by simulating the application of MSSR to enhance the resolution of an image of two elongated nanoscale objects at an angle, in the presence and absence of constructive or destructive interference. We observe significant resolution enhancement in all three cases, with the caveat that the CSR techniques used in this work are not able to adjust the intensity of features within the image to compensate for constructive and destructive interference. Even with this limitation, we demonstrate that CSR can be used to enhance the resolution of experimental harmonic generation microscopy images by up to 3.4× for isolated nanostructures and up to 2×, in more complicated samples. We additionally show that SRRF and DPR are able to preserve the polarization dependence of the SHG signal, thereby enabling super-resolution polarization-resolved SHG microscopy. These results are obtained without any modification to the microscope, and little modification to the imaging procedure, and therefore present a much lower barrier to entry than other super-resolution nonlinear optical imaging techniques.

## Materials and methods

### Sample preparation

ZnSe nanowires were grown by the vapor-liquid-solid growth method^18^ on a silicon wafer. Once cooled, the wafer is deposited in water, and sonication is applied separating the nanowires from the substrate. A drop of a solution containing nanowires was deposited on a cover slip and allowed to dry leaving nanowires on the glass surface. A cover slip was then placed on top. Cubic BaTiO_3_ nanoparticles (100 nm diameter, US Research Nanomaterials Inc.) were deposited between two cover slips (VWR, 16004-348). Starch particles were extracted from fresh maize obtained from a local market by crushing with a pestle and mortar in distilled water and centrifuging the resultant suspension at 5000 g for 4 min similar to Cisek et al.^19^. The purified starch was subsequently diluted, and a drop was deposited on a cover slip containing a parafilm spacer, covered with a cover slip, and imaged before the sample dried. ~10 μm thick sections of formalin fixed bovine lateral digital extensor tendon were prepared as described in Harvey et al.^20^.

### SHG and THG imaging

SHG and THG intensity imaging and polarization-in polarization-out (PIPO) SHG imaging was performed using our homebuilt laser scanning nonlinear optical microscope and analysis framework previously described^21^. To reduce the risk of artifacts resulting from external vibrations the laser and microscope setup are mounted to a vibration dampening optical table (T1540R, Thorlabs). The excitation laser (Femtolux 3, Ekspla) has a wavelength of 1030 nm, pulse duration of ~ 290 fs, and repetition rate of 5 MHz. The average power at the sample was in the range of 0.3–1.6 mW (peak power ~ 200–1000 W), which gives an average power density at the focal spot of ~ 60–250 kWcm^− 2^. Images with 100 × 100 or 200 × 200 pixels are obtained with a pixel size of 85–190 nm (only for the starch and tendon images shown in Fig. 3), and a pixel dwell time of 12 µs. A 0.8 NA air immersion objective (Plan-Apochromat 20×, Carl Zeiss AG) and a 0.85 NA objective (custom, Omex Technologies, Inc) are used for excitation and collection of transmitted signals, respectively. Bandpass interference filters centered at 515 nm (65–153, Edmund Optics Inc) and 340 nm (65–129, Edmund Optics Inc) are used to separate SHG and THG signal from the excitation beam. Detection is performed using a photomultiplier tube detector (H10682-210, Hamamatsu Photonics K.K.) in photon counting mode. The excitation laser power is set such that the maximum intensity per pixel is ~ 24 counts per pixel per frame, corresponding to 2 MHz. For intensity imaging we utilize either linearly polarized excitation with the angle selected to obtain optimal SHG intensity for the BaTiO_3_, collagen, and ZnSe samples, or circularly polarized excitation for the starch particle to obtain uniform SHG signal across its radial structure. SHG intensity images are saved as stacks of 500 individual frames to provide the temporal information needed for CSR processing.

### PIPO SHG imaging

For PIPO SHG imaging we obtain stacks of 50 frames of SHG intensity ($\it \:\mathrm{I}$) at all combinations of 8 polarization angles ($\it \:{\uptheta\:}$) in excitation, and 8 angles ($\it \:\phi\:$) of a linear polarizing filter (analyzer) in the collection path. The laser power is set such that the SHG intensity within the images across all angles is ~ 24 counts per pixel per frame, or a total of 1200 counts. At the end of the imaging process a final 50 frames are obtained at the initial values of $\it \:{\uptheta\:}$ and $\:\phi\:$ to verify that there is no movement or damage of the sample during imaging, and to provide a resolution reference for CSR analysis (see below). Both the raw and CSR processed polarization data is then fit to the following equation using a custom MATLAB (The MathWorks, Inc) program to obtain $\it \:{\uprho\:}$, a commonly reported structural parameter for PSHG microscopy.

1 $$\:\mathrm{I}=\mathrm{A}{\left|{\uprho\:}{\mathrm{cos}}^{2}\left({\uptheta\:}^{\prime\:}\right)\mathrm{cos}\left(\phi\:^{\prime\:}\right)+{\mathrm{sin}}^{2}\left({\uptheta\:}^{\prime\:}\right)\mathrm{cos}\left(\phi\:^{\prime\:}\right)+\mathrm{sin}\left(2{\uptheta\:}^{\prime\:}\right)\mathrm{sin}\left(\phi\:^{\prime\:}\right)\right|}^{2}+\mathrm{F}$$

Here $\:{\theta\:}^{{\prime\:}}=\theta\:-\delta\:$ and $\:{\phi\:}^{{\prime\:}}=\phi\:-\delta\:$, and $\it \:{\updelta\:}$ is the angle of the structure under investigation within the image plane with respect to the polarization orientation of the excitation laser at $\it \:{\uptheta\:}$ = 0, $\it \:\mathrm{A}$ is the constant of proportionality between the square of the SHG field and the number of counts measured on the detector, and $\it \:\mathrm{F}$ is the noise. This equation is valid for cylindrically symmetric assemblies of uniaxial SHG emitters (e.g. the peptide bonds in a collagen triple helix^22^). A more general symmetry (e.g. trigonal^23)^ may yield more details about higher order organization within a sample, however, for the highly aligned collagen in tendons, which will be imaged here, Eq. (1) should still be applicable.

### SRRF processing

SRRF analysis is performed using code which is publicly available as part of the NanoPyx package^24^. A full description of SRRF, and evaluation of its performance for various fluorescence modalities can be found in Gustafsson et al.^15^. Briefly, SRRF assumes that the image is formed from point sources convolved with a radially symmetric point spread function (PSF), and therefore the degree of local radial symmetry (radiality) can be used to enable localization beyond the diffraction limit. To obtain super-resolution, each pixel of the image is first split into a user defined number of sub pixels (the magnification parameter), the radiality over a user defined ring radius is then determined at each pixel of the image. Since noise may also lead to large radiality values, SRRF is usually performed on several consecutive frames of intensity data, and analysis of the radiality over time is performed to reject features which are uncorrelated in time. Here the radiality average over multiple frames (500 for SHG and THG intensity data, and 50 for PIPO SHG) is used to produce the super-resolved image. In general, a higher magnification and smaller ring radius will lead to higher resolution, however care must be taken to obtain optimal resolution without inducing artifacts.

Here the optimal magnification and ring radius is determined by performing SRRF analysis separately on the first and second half of the intensity stack for intensity data, or the first and last PIPO SHG image stacks containing 50 frames each (which are obtained at the same polarization conditions). This results in two presumably identical super-resolved images. A Fourier ring correlation (FRC)^25^ is then performed in Fiji^26^ to obtain the correlation between the two images as a function of spatial frequency, and the highest frequency at which the correlation is greater than 1/7 is reported as the resolution^27^. The magnification and ring radius is selected to optimize the resolution obtained using an FRC, and SRRF analysis of the entire intensity stack, or all PIPO SHG polarization states is then performed using those input parameters. The parameters used for SRRF processing, and for the other CSR techniques used here, can be found in tables S1-S3 in the supplement.

### MSSR processing

MSSR utilizes mean shift theory to shift intensity towards the local maximum within a region determined by the size of imaging PSF^16^. This procedure can be performed iteratively to further enhance the resolution of an image (termed MSSR^0^, MSSR^1^, etc…). The user provided parameters are the FWHM of the PSF of the imaging system, a magnification parameter similar to SRRF, and the desired number of iterations. MSSR is performed using a Fiji plugin described in^16^ and the magnification and number of iterations are selected to maximize the resolution determined using an FRC as described above. The FWHM of the imaging PSF is calculated using the following equation^28^.

2 $$\:\mathrm{F}\mathrm{W}\mathrm{H}\mathrm{M}\approx\:\frac{0.61{\uplambda\:}}{\sqrt{\mathrm{n}}\mathrm{N}\mathrm{A}}$$

Here λ is the wavelength of the excitation laser (1030 nm here), NA is the numerical aperture of the excitation objective (0.8 here), and n is the order of the nonlinear process, 2 for SHG and 3 for THG. It is important to note that this equation is an approximation which differs from the true FWHM by ~ 15%. We utilize it here because an estimate of the PSF size is sufficient for both MSSR and DPR, and we have verified for all MSSR and DPR images shown in Figs. 3 and 5 that a ± 15% change in the estimated FWHM yields nearly identical super-resolution images (Pearson correlation coefficient > 0.99).

One advantageous feature of MSSR is that it can be used to further enhance the resolution of images which have already undergone super-resolution processing, therefore in addition to applying MSSR to our raw images, we also apply it to SRRF processed images as has been previously demonstrated^16^. Similar to the approach above to decrease the effect of noise MSSR is performed on a sequence of consecutive frames (500 for SHG and THG intensity images, 50 for PIPO SHG), and the mean is presented as the super-resolution reconstruction.

### DPR processing

In DPR the image is first magnified such that the pixel size is one eighth of the FWHM of the imaging PSF. The intensity is then reassigned between pixels based on the direction and magnitude of local intensity gradients. We perform DPR processing using publicly available MATLAB code^17^. The two input parameters for DPR are the FWHM of the imaging PSF, which is determined using Eq. (2), and the gain setting which is used to determine how the intensity gradients are weighted, which is selected to minimize the FRC resolution as above. As with SRRF and MSSR the effect of noise is reduced through temporal analysis. DPR processing is performed on a sequence of consecutive frames similar to SRRF and MSSR, and the mean of the DPR processed frames is used as the super-resolution image.

### Quantifying image quality

To quantify the accuracy of CSR reconstructions we utilize NanoJ-SQUIRREL^29^. SQUIRREL analysis determines the PSF, which when convolved with a CSR image optimally reproduces the raw image. This analysis provides two parameters which are often used for quantifying the performance of CSR methods, the resolution-scaled Pearson coefficient (RSP) which is the Pearson correlation coefficient between the raw image and the resolution-scaled super-resolution image, and the resolution-scaled error (RSE) which is the root-mean-square error between the raw image and the resolution-scaled super-resolution image. Here we normalize the raw images to the range 0–100 prior to SQUIRREL analysis, therefore the RSE parameter represents the percent root-mean-square error between the super-resolution and raw SHG images. SQUIRREL also allows spatial mapping of the RSE parameter, and we provide error maps for all the super-resolution images presented here in the supplement. To provide a benchmark for “perfect” super-resolution reconstructions, and to demonstrate that the images used here are not highly affected by sample drift or damage, we perform SQUIRREL analysis of the raw images by comparing the sum of the first and last 250 frames of the SHG and THG intensity stacks, or first and last 50 frames of the PIPO SHG stacks, treating the first half of the stack as an image, and the second half as a super-resolution reconstruction. We use this as a convenient method of demonstrating that the sample is unchanged during the imaging process, and that no processing of the image stacks (e.g. drift correction) is required before CSR analysis. This is of particular importance since we are using the FRC as a resolution metric, which requires that the structure under investigation is unchanged during the imaging process.

### Theoretical results

To begin with it is important to discuss the applicability of CSR to harmonic generation imaging. Each of these CSR techniques assumes that an image $\:\left(\mathrm{h}\right)$ can be described as the convolution of a collection of point sources termed the object, $\:\mathrm{O}$, with a point spread function (PSF), i.e. $\:\mathrm{h}={\left|\mathrm{p}\mathrm{s}\mathrm{f}\right|}^{2}\mathrm{*}{\mathrm{O}}^{2}$. This is true for incoherent processes such as fluorescence; however, it is not generally true for coherent processes such as SHG and THG, in which constructive or destructive interference between individual point sources results in a more ambiguous relationship between the object and image, i.e. $\:\mathrm{h}=|{\left.\mathrm{p}\mathrm{s}\mathrm{f}\mathrm{*}\mathrm{O}\text{}\right|}^{2}$^28^. However, when the object in question is a point source (or a collection of point sources spaced further than the PSF width) then the two descriptions become equivalent. Since harmonic generation microscopy is often applied to samples containing isolated nanostructures (e.g. nanowires) or collections of nanostructures (e.g. collagen fibrils in tissue sections), which have sizes much smaller than the typical PSF size of an optical microscope, harmonic generation imaging of these samples will in many cases likely produce results similar to those which would be obtained with an incoherent imaging process, and therefore CSR may be applicable to improve localization in harmonic generation microscopy.

**Fig. 1:**
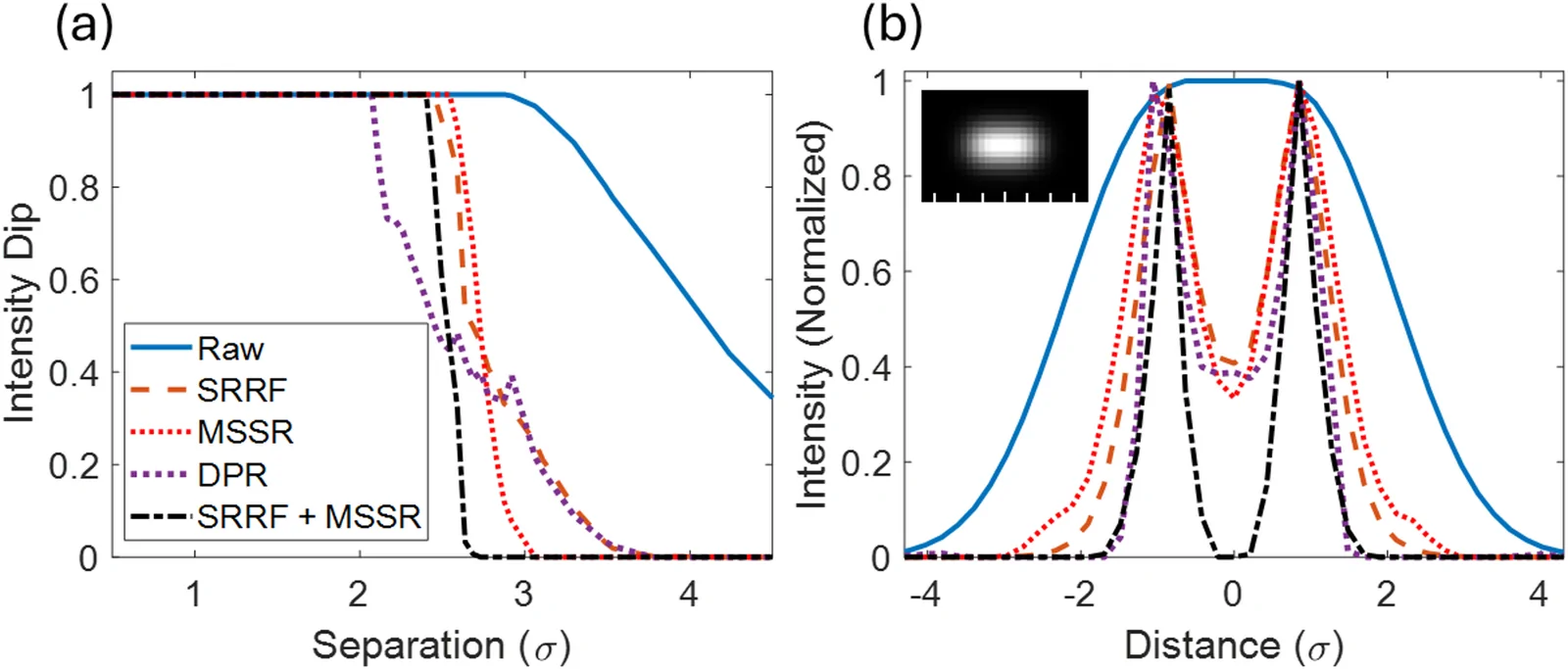
CSR processing on simulated point emitters. A plot (a) of the intensity dip between two constructively interfering point emitters as a function of their separation distance (in units of the standard deviation of the PSF $\:{\upsigma\:}$), and an intensity profile for the inset image of two point emitters separated by 2.8$\:\sigma\:$ (b). The intensity dip is the minimum value of a normalized line profile between the two points, and the ticks in the inset image are separated by 1$\:{\upsigma\:}$. The CSR parameters used can be found in supplementary tables S1 – S3.

To evaluate the applicability of CSR techniques to coherent nonlinear microscopy we perform CSR processing of simulated microscopy images. Figure 1a summarizes results obtained for simulations of identical constructively interfering point emitters at various distances. The excitation PSF used is a Gaussian function with standard deviation $\:{\upsigma\:}$. The emitters are separated by a variable distance defined in units of $\:{\upsigma\:}$ and we report the minimum intensity between them. These results show that CSR processing provides significant resolution enhancement compared to the raw images, i.e. a dip in the intensity profile between the two emitters can be seen at lower separation distances after CSR processing. An example of this can be seen for the line profile of two emitters 2.8 $\:{\upsigma\:}$ apart as shown in Fig. 1b and its inset, where the emitters are not resolved in the raw image (there is no dip in the intensity profile), but all of the CSR techniques investigated here are able to resolve the two points. For the worst performing CSR technique (MSSR) we obtain a ~ 1.1$\:\times\:$ enhancement in the Sparrow limit (the distance at which intensity begins to dip) and a ~ 1.3$\:\times\:$ enhancement in the Rayleigh limit (26.3% intensity dip), and the best performing technique (DPR) providing a ~ 1.4$\:\times\:$ enhancement in the Sparrow limit and a ~ 1.7$\:\times\:$ enhancement in the Rayleigh limit compared to the raw images. One important difference between coherent and incoherent imaging which must be considered is that the resolution in coherent imaging is object dependent. In Fig. 1 we show results for constructively interfering emitters, which leads to the lowest possible resolution, two noninterfering emitters, equivalent to the incoherent case will enable slightly higher resolution (see for example a similar figure for the incoherent case in^16^, and two destructively interfering emitters will always show an intensity dip, however the total intensity will be greatly reduced if they are in close proximity. Further images showing resolution enhancement for each technique in the cases of incoherent imaging, constructive, and destructive interference can be found in Figure S1 in the supplement.

The major flaw of currently available CSR techniques for use in coherent nonlinear microscopy is that they do not adjust the intensity of features within the image to compensate for constructive and destructive interference. Therefore, while these CSR techniques may help to improve the localization of nanoscale objects, the relative intensity of different regions within an image is not necessarily correlated with the relative density of emitters, as is already the case for coherent nonlinear microscopy. To our knowledge the only currently available super-resolution nonlinear microscopy techniques which are able to correct for this effect are coherent image scanning microscopy which requires interferometric contrast to determine the relative phase at different locations within an image^7^, or techniques which physically reduce the size of the excitation PSF, by using a photonic nanojet for example^9^. The development of new CSR techniques specialized for coherent imaging processes is an exciting avenue for future development which may provide more accurate reconstructions of the underlying structures based on SHG and THG images.

### Experimental results

**Fig. 2:**
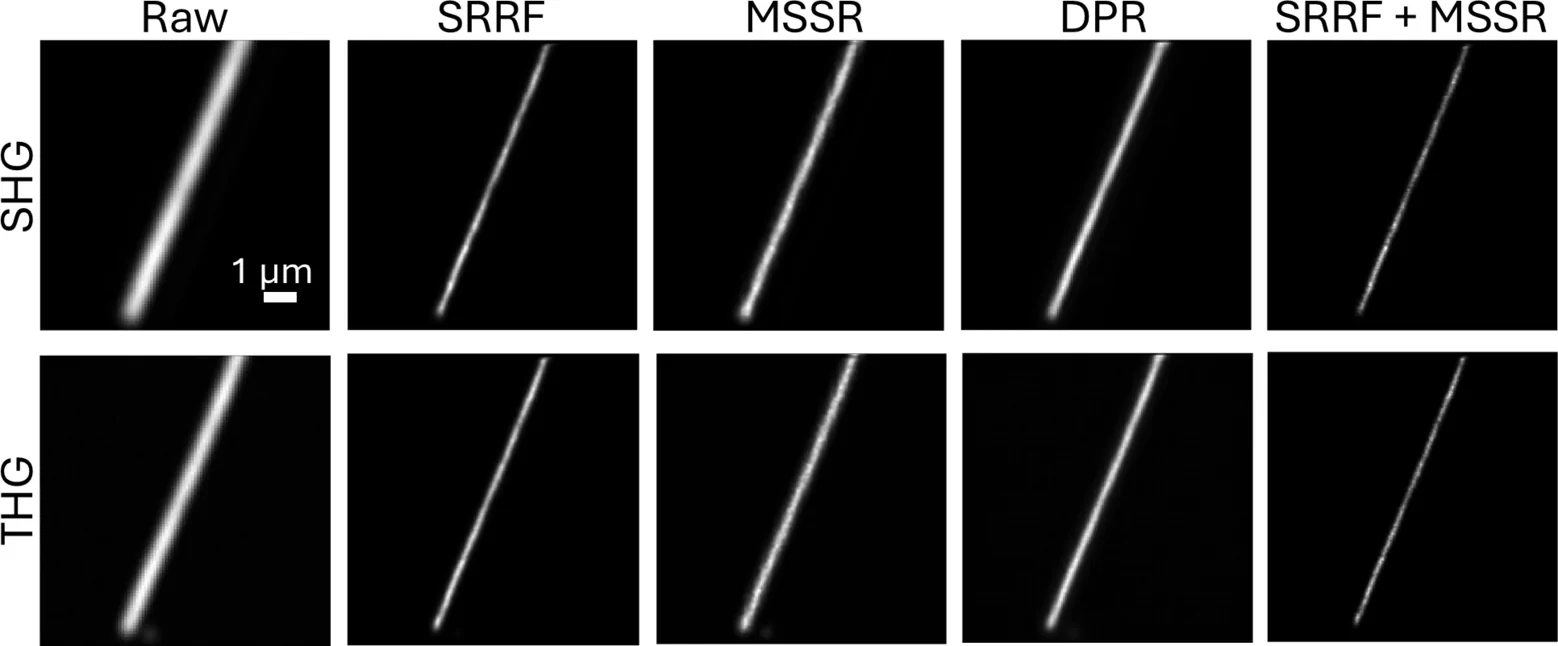
SHG and THG images of a ZnSe nanowire after a variety of CSR procedures. The scale bar applies to all images. Intensity profiles across all images can be found in Fig. S3 in the supplement, and the CSR parameters used can be found in tables S1 – S3.

Now we attempt to gain a benchmark for the resolution enhancement which is experimentally achievable by each super-resolution technique by taking SHG and THG intensity images of ZnSe nanowires. These nanowires have typical diameters < 200 nm, much smaller that the ~ 555 nm and ~ 453 nm theoretical FWHM of the PSF for SHG and THG for the microscope used here (see Eq. (2)). Therefore, these images allow direct measurements of the effective PSF FWHM after super-resolution processing. This is obtained by fitting a Gaussian function to intensity profiles across five different areas of the images, and the mean FWHM is reported with the range reported as uncertainty. These results, as presented in Fig. 2; Table 1 demonstrate that for isolated nanostructures CSR processing is able to significantly enhance the lateral resolution of nonlinear microscopy images. From this we see that combining SRRF and MSSR provides the best resolution enhancement of the techniques used here with the SHG and THG images having a FWHM resolution 3.2–3.4 times greater than the diffraction limit. This is quite significant as to our knowledge the best reported resolution enhancement for CSR of SHG images using deconvolution is ~ 1.3×^13^ and the best reported resolution enhancement for hardware based super-resolution approaches is ~ 2.5×^30^. SRRF processing on its own is able to achieve the second highest resolution with an enhancement of 2.3–2.4×, DPR and MSSR achieve fairly similar resolution enhancements in the range of 1.5× – 1.8×.

**Table 1:** Resolution enhancement in a ZnSe nanowire using CSR. The FWHM is obtained for 5 points along the nanowire, and the mean ± range is reported here.

Processing	FWHM SHG	FWHM THG	RSP SHG	RSE SHG	RSP THG	RSE THG
Raw	513 ± 6 nm	433 ± 13 nm	0.999	0.8%	0.995	1.7%
SRRF	212 ± 11 nm	187 ± 10 nm	0.980	3.7%	0.979	3.6%
MSSR	286 ± 29 nm	278 ± 43 nm	0.991	2.4%	0.991	2.3%
DPR	286 ± 16 nm	244 ± 15 nm	0.997	1.5%	0.998	1.1%
SRRF + MSSR	149 ± 20 nm	135 ± 12 nm	0.969	4.7%	0.973	4.1%

While measurement of the intensity profile across individual nanostructures is a convenient method for quantifying the maximum achievable resolution enhancement in super-resolution microscopy and has been reported for many of the previous proposed super-resolution harmonic generation imaging techniques^6,8,10,30^ since the local resolution in harmonic generation imaging is dependent on the sample structure (see above) this is not a particularly effective way of quantifying resolution. We therefore perform SHG intensity imaging of a cluster of BaTiO_3_ nanoparticles, a starch particle, and densely packed collagen fibrils within a section of tendon and utilize the FRC to determine resolution. These samples have more complicated structures than the individual nanowires presented above and therefore present a good opportunity to assess the resolution enhancement in complex samples, and to investigate the quality of our super-resolution reconstructions. From the FRC resolution (see Materials and Methods) presented in Table 2 DPR provides the best resolution enhancement (1.8–2.0$\:\times\:$) for both the nanoparticles and the tendon section, SRRF and SRRF + MSSR provide slightly worse results (1.4–1.5$\:\times\:$), and MSSR provides only 1.1–1.2$\:\times\:$ resolution enhancement. For the starch particle all techniques provide relatively low-resolution enhancement according to the FRC, likely because the starch has a relatively uniform structure without higher frequency features that can be resolved.

**Fig. 3:**
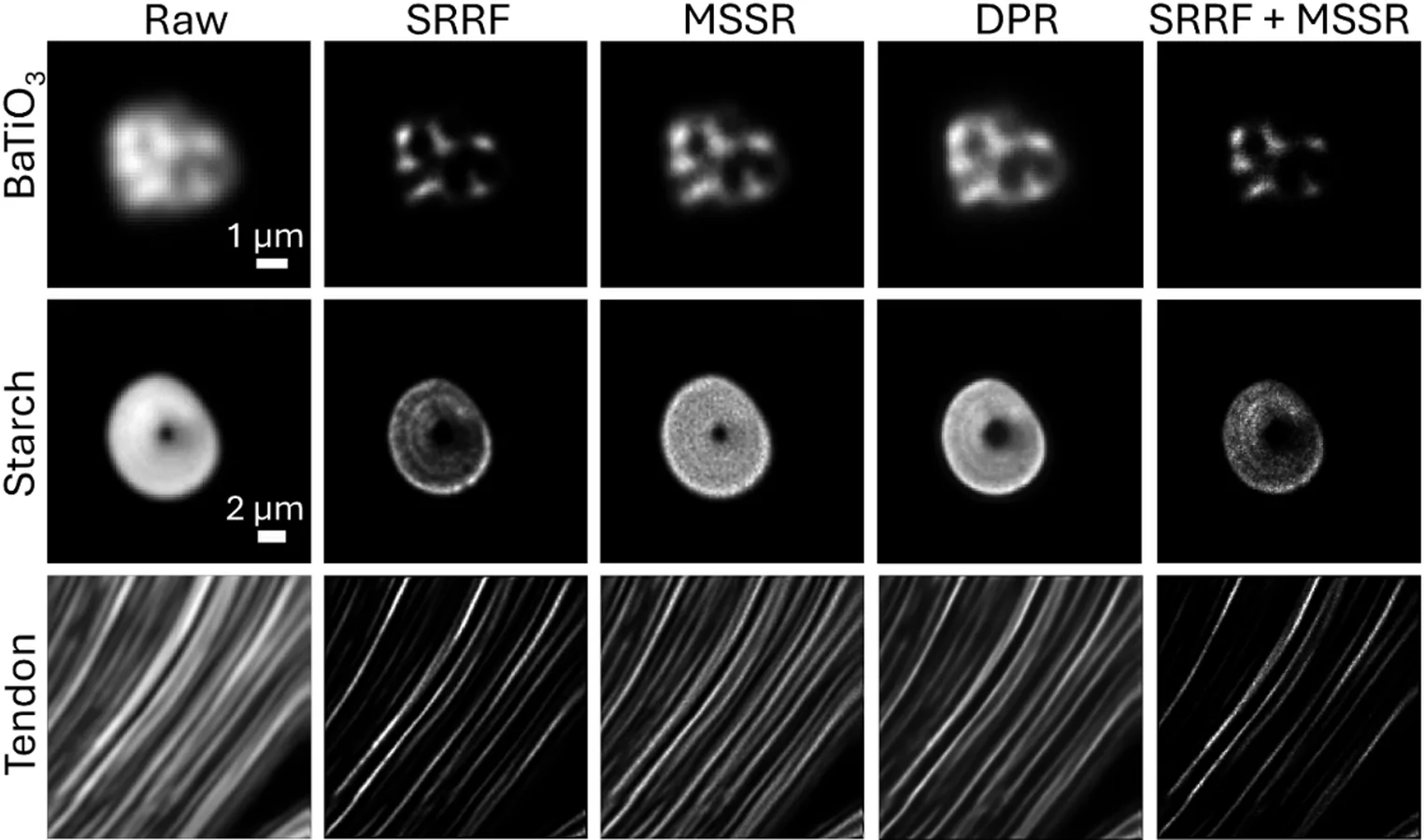
Super-resolution processing of SHG images of a cluster of BaTiO_3_ nanoparticles (first row), a starch particle (second row), and a section of tendon (third row). The scale bar in the starch image also applies to the tendon images. Intensity profiles across selected regions of each sample can be found in Fig. S5 in the supplement and the CSR parameters used can be found in tables S1 – S3.

From a more qualitative standpoint the sharpening of the effective PSF provided by these image processing techniques does provide increased clarity to the images presented in Fig. 3. In the cluster of BaTiO_3_ nanoparticles each of the CSR techniques are clearly able to better resolve the six bright spots present within the image than the raw SHG intensity image. In the starch images SRRF, DPR, and SRRF + MSSR reveal several concentric rings, which are likely starch growth rings composed of alternating semi-crystalline and amorphous shells^31^. While SHG has found extensive application in characterizing the structure of starch particles^32^ these growth rings are not typically resolved by SHG. In the raw tendon image, the imaging PSF causes the densely packed collagen fibrils to blur together, however CSR processing, particularly SRRF and SRRF + MSSR allows individual fibrils to be clearly discriminated.

**Table 2:** Resolution and SQUIRREL parameters for the images presented in Fig. 3.

Processing	BaTiO_3_			Starch			Tendon		
	FRC	RSP	RSE	FRC	RSP	RSE	FRC	RSP	RSE
Raw	294 nm	1.000	0.6%	490 nm	1.000	0.7%	282 nm	0.997	1.5%
SRRF	203 nm	0.982	4.6%	399 nm	0.976	5.8%	197 nm	0.933	6.9%
MSSR	261 nm	0.994	2.6%	506 nm	0.989	3.8%	233 nm	0.968	4.7%
DPR	164 nm	0.998	1.5%	454 nm	0.996	2.3%	144 nm	0.984	3.3%
SRRF + MSSR	192 nm	0.956	7.1%	425 nm	0.965	7.0%	200 nm	0.861	9.6%

By looking at the SQUIRREL parameters presented in Tables 1 and 2 we can get an idea of the relative quality of each of the CSR techniques used here. For the RSP values we see a minimum value of 0.861 for the SRRF + MSSR reconstruction of tendon shown in Fig. 3, with all other images having a RSP value greater than 0.956. This indicates that all of the CSR reconstructions presented here have very good correlations with the raw SHG and THG images, demonstrating the high fidelity of each of these processes. In all cases presented above DPR consistently produces the highest RSP values with a mean of 0.995, indicating a near perfect correlation with the raw intensity images. MSSR (0.987) and SRRF (0.970) are consistently second and third best, and SRRF + MSSR consistently produces the lowest RSP values (0.945), which is expected as this form of processing compounds all the errors introduced by both SRRF and MSSR.

The RSE parameter tells a similar story with DPR consistently producing the lowest RSE values (mean 1.9%), MSSR second lowest (3.2%), SRRF third (4.9%) and SRRF + MSSR highest (6.5%). The images shown in Fig. 3 provide good demonstrations of a few of the kinds of errors often associated with CSR images. For example, the SRRF image of starch shows a clear “hollowing out” artifact in which the edges of the starch (particularly in the lower right) have disproportionately high intensity compared to the interior, which is a well-known error with CSR techniques^17^(this can also be seen as locations of large error in Fig. S3 in the supplement). The SRRF and SRRF + MSSR, and to a much lesser extent the MSSR images of tendon shown in Fig. 3 also show a large amount of nonlinearity in the CSR reconstruction, meaning that there is a lack of correlation between relative intensities within the raw image and the CSR reconstruction. These images clearly show that these CSR processes arbitrarily prioritize certain fibrils over others and introduce nonuniformity along individual fibrils which are not present in the raw image (this can be seen very clearly in Fig. S6 in the supporting information). DPR is able to mostly avoid the two types of CSR artifacts which we have highlighted here, as DPR moves intensity between pixels based on intensity gradients, therefore preserving the relative intensity between different areas of an image.

**Fig. 4:**
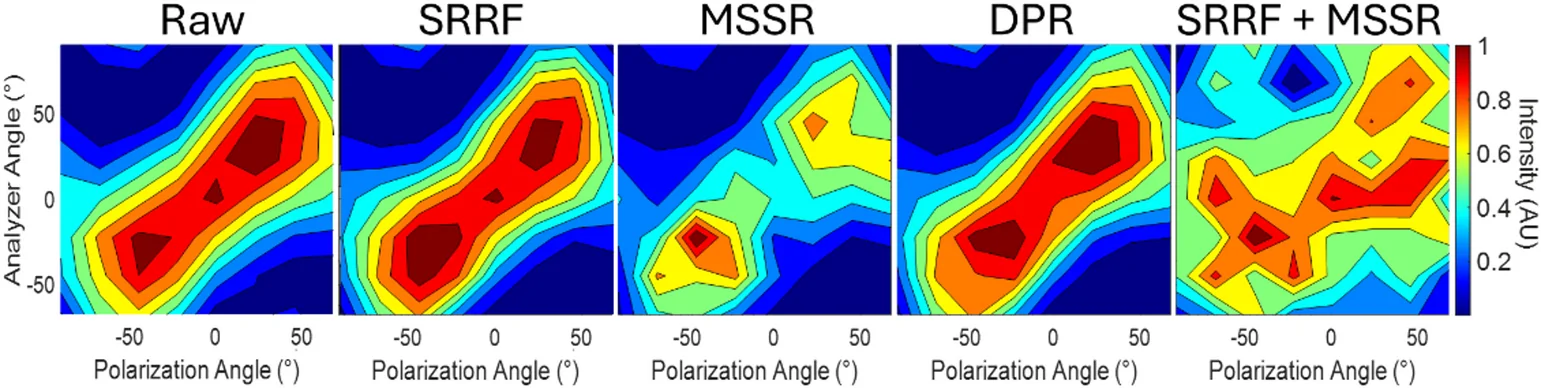
Contour plots of measured SHG intensity for each excitation polarization and analyzer angle (each over a 180° range) for a representative pixel in the raw image and after super-resolution processing.

**Fig. 5:**
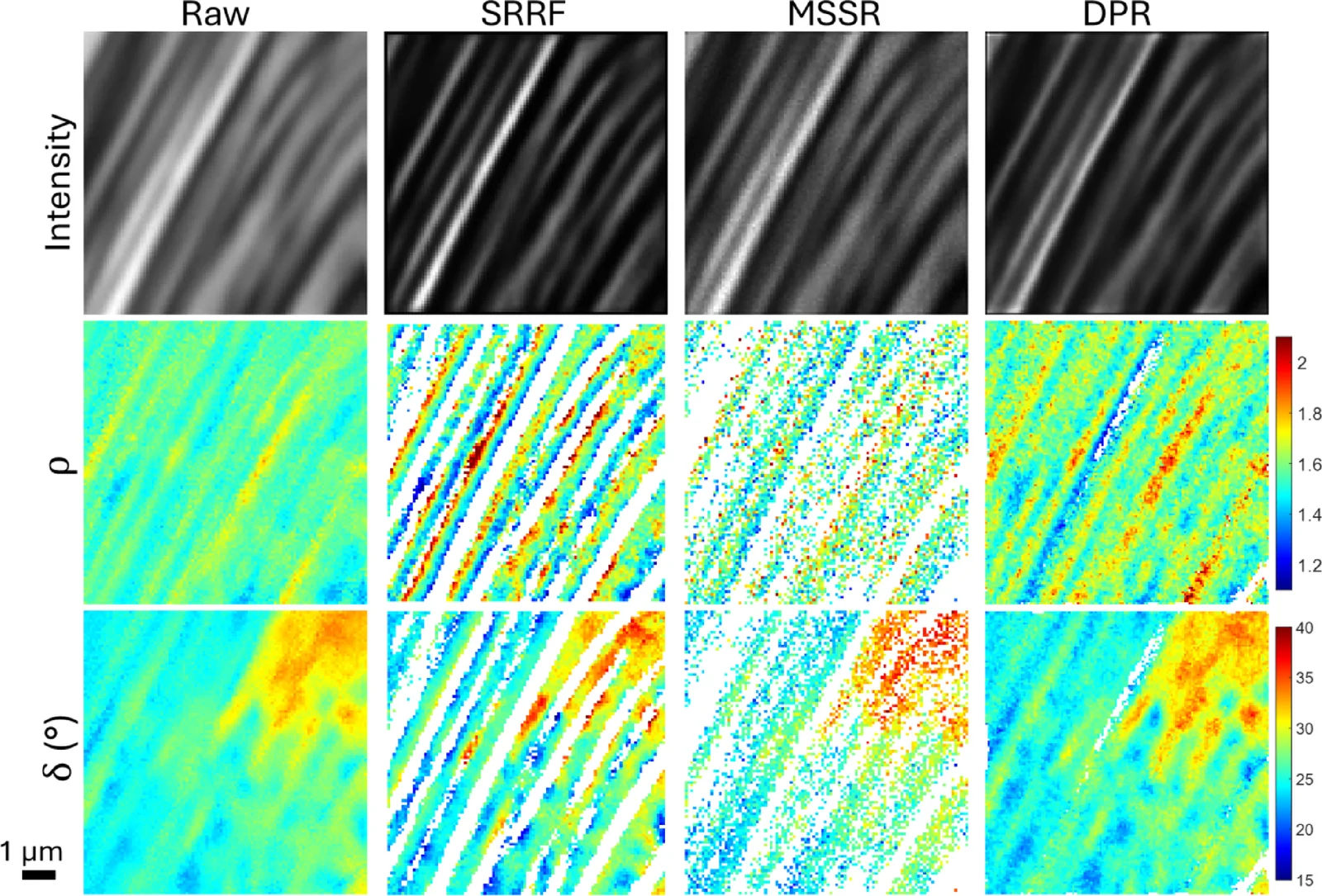
SHG intensity (obtained by summing the intensities of the 64 polarization conditions in PIPO SHG), and images of the $\it \:{\uprho\:}$ and $\it \:{\updelta\:}$ parameters for PIPO SHG data after super-resolution processing. $\it \:{\updelta\:}$ is measured clockwise from the vertical axis of the image. The FRC resolution is 305 nm for the raw image, 230 nm for SRRF, 336 nm for MSSR, and 267 nm for DPR, the RSP values are 0.986, 0.944, 0.995, and 0.979, and the RSE values are 2.7%, 5.3%, 1.6%, and 3.3%. The CSR parameters used for each image can be found in supplementary table S1 – S3.

In addition to investigating the potential of CSR to enhance the resolution of SHG and THG intensity images we also investigate the application of CSR for polarization-resolved SHG imaging. To do this we acquire PIPO SHG images of collagen fibrils within a tendon section. Figure 4 shows contour plots of SHG intensity as a function of the excitation polarization and analyzer angles for the same pixel of a raw PIPO SHG image, and the same image after several CSR processes. From this it is clear that SRRF and DPR both do a fairly good job at reproducing the polarization dependence of SHG signal, as the contour plots for each of these processes have shapes strongly resembling that in the plot for the raw data. In contrast the MSSR and SRRF + MSSR plots do not accurately reproduce the polarization dependence of SHG. By fitting the data at each pixel of our super-resolved PIPO SHG data using Eq. (1) we are able to obtain super-resolved maps of the *ρ* parameter for these samples as shown in Fig. 5. From this we obtain mean values of *ρ* of 1.56 ± 0.05 (mean ± standard deviation) for the raw image, 1.6 ± 0.2 for the SRRF image, 1.6 ± 0.1 for MSSR, and 1.6 ± 0.1 for DPR. These values are within the range previously measured^33^ for collagen fibrils isolated from extensor tendons. All of the processing conditions are also able to provide fairly consistent $\it \:{\updelta\:}$ angles which appear to be in good alignment with the local fibril direction to within a few degrees. For the raw and DPR images nearly every pixel within the frame has adequate SHG intensity to enable a high quality ($\:{\mathrm{R}}^{2}$ > 0.8) fit using Eq. (1), while the SRRF processed image has adequate resolution to enable individual fibrils to be clearly seen in the *ρ* map. For MSSR many of the pixels can be fit with similar $\it \:{\uprho\:}$ and $\it \:{\updelta\:}$ values to the other images, however the pixels within the MSSR image fit much less consistently than for other techniques. The mean R^2^ for pixels above the $\:{\mathrm{R}}^{2}$ > 0.8 threshold is 0.98 for the raw image, 0.90 for SRRF, 0.84 for MSSR, and 0.93 for DPR. For SRRF + MSSR no pixels fit with $\:{\mathrm{R}}^{2}$ > 0.8, and this data is therefore not shown in Fig. 5. Notably, many of the fibrils in the SRRF processed *ρ* map show a gradient in *ρ* values going from high *ρ* on the left to low *ρ* on the right. This gradient has previously been observed in individual isolated collagen fibrils and occurs as a result of nonuniformities in the focal field polarization due to high numerical aperture focusing^33^, and its appearance in the SRRF processed image is strong evidence that SRRF is able to isolate the SHG intensity from individual fibrils within a densely packed tissue. This is a potentially exciting result as polarization-resolved SHG imaging has been shown to have applications in the automated diagnosis of diseases such as cancers based on changes in collagen organization within the extracellular matrix^34^. As we have shown, the application of CSR techniques to PSHG imaging can provide a clearer picture of collagen organization within tissue and could thereby enable earlier and more accurate disease diagnosis.

## Discussion

We now discuss the relative strengths and weaknesses of the CSR techniques investigated. DPR consistently produces the highest quality images as measured using both the RSP and RSE. It can enhance the resolution of nonlinear microscopy images by ~ 1.7–2× (as determined from FRC values above) comparable to the best reported results using deconvolution-based CSR techniques and preserves the polarization dependence of SHG signal. DPR would therefore be the best choice for general SHG/THG intensity imaging. However, SRRF enables higher resolution than DPR for PIPO SHG imaging while still preserving the polarization dependence of SHG signal, albeit with lower quality reconstructions. Our results suggest that MSSR on its own is not particularly useful for super-resolution nonlinear optical imaging, as it enables only slight increases in resolution at slightly lower quality than DPR, and does not consistently preserve the polarization dependence of SHG signal. However, when used to further enhance the resolution of SRRF images of isolated nanostructures we achieve resolution enhancements of up to 3.4×, which far exceeds the best reported resolution enhancements of SHG or THG images in the literature, albeit with lower quality reconstructions than the other techniques investigated here.

We obtained these images with very little modification to our typical imaging procedure, with the only significant change being the requirement to save image stacks containing sequences of frames, rather than only saving the sum of the frames. This means that there is no significant difference in the experimental complexity or imaging speed for CSR compared to standard nonlinear optical imaging. The use of an imaging system which enables the collection of large numbers of frames at high speed, such as a wide field microscope, may however enable higher resolution and better-quality CSR reconstruction. All software used here for CSR processing is publicly available and easy to use^16,17,24^. Using CSR to enhance the resolution of nonlinear microscopy images therefore presents a major advantage in terms of simplicity compared to hardware based super-resolution techniques.

One major disadvantage of these CSR techniques for super-resolution nonlinear optical imaging is that they do not enhance axial resolution, while other techniques have shown axial resolution enhancement of up to 1.5×^10^. However, other CSR techniques (e.g. eSRRF^35)^ can enhance the axial resolution of volumetric data. Therefore, the use of multiphoton techniques such as SHG, THG, or multiphoton excitation fluorescence^36^, which already have high axial resolution compared to single photon imaging techniques, for super-resolution volumetric imaging presents an exciting opportunity for development. This is of particular interest since there have been significant recent advancements in utilizing PSHG to image deep within collagen containing tissues^37–39^ and CSR therefore presents opportunities to obtain high resolution PSHG data for large volumes of intact tissue.

## Conclusions

We compare four procedures for CSR of SHG and THG images and demonstrate that CSR techniques are able to provide a resolution enhancement of up to 3.4× compared to the diffraction limit when imaging isolated nanostructures. This is a much larger resolution enhancement than has been previously reported for super-resolution SHG/THG imaging and far surpasses the resolution enhancement previously achieved using deconvolution-based CSR techniques. Additionally, we find that SRRF and DPR are able to preserve the polarization dependence of SHG signal, thereby enabling super-resolution polarization-resolved SHG microscopy. Application of these CSR techniques requires no modification to the microscope system used, little modification to imaging procedures, and make use of publicly available software therefore presenting a lower barrier to entry compared to hardware-based super-resolution techniques. These results therefore demonstrate that the application of CSR to SHG/THG imaging can lead to exciting opportunities for future applications of super-resolution nonlinear microscopy in biomedical or material science research.

## Supplementary Information

Below is the link to the electronic supplementary material.

Supplementary Material 1

## Data Availability

Data underlying the results presented in this paper are available in figshare at https://figshare.com/s/1e1c1cb11e61642e52ae.
