# Supplementary material for: Evaluating the applicability of computational super-resolution techniques for harmonic generation microscopy
Source: Sci Rep. 2026 Jun 23;16:28839. doi: 10.1038/s41598-026-58983-0 (PMC13578285; doi:10.1038/s41598-026-58983-0)
Supplement: Supplementary file 1 [file 41598_2026_58983_MOESM1_ESM.docx]

Evaluating the Applicability of Computational Super-Resolution Techniques for Harmonic Generation Microscopy: Supplement

MacAulay Harvey^1^, Richard Cisek^1^, Sarry Al-Turk^2^, Harry. E. Ruda^2^, Laurent Kreplak^3,†^, and Danielle Tokarz^1,*^

^1^Department of Chemistry, Saint Mary’s University, 923 Robie Street, Halifax, NS, B3H 3C3, Canada

^2^Centre for Advanced Nanotechnology, University of Toronto, 170 College Street, Toronto, Ontario M5S 3E4, Canada, Department of Materials Science and Engineering, University of Toronto, 184 College Street, Toronto, Ontario M5S 3E4, Canada, Department of Electrical and Computer Engineering, University of Toronto, 10 Kings College Road, Toronto, Ontario M5S 3G4, Canada

^3^Department of Physics and Atmospheric Science and School of Biomedical Engineering, Dalhousie University, Halifax, NS, B3H 4J5, Canada

^†^kreplak@dal.ca

^*^danielle.tokarz@smu.ca

Figure S1. CSR processing of simulated microscopy images of a structure consisting of two line objects at an angle. Simulations are carried out for an incoherent imaging process and for a coherent imaging process in which the two objects have the same orientation and therefore interfere constructively, and opposite orientation and interfere destructively. The FWHM of the Gaussian PSF used in the simulations is 500 nm. The point at which the two line objects are resolved according to the Rayleigh limit is shown by a green dotted line in the incoherent and constructive images. All raw images have the true locations of the line objects superimposed in red. Note that the intensity scale of the six images is independently adjusted for visualization.

Figure S2. SQUIRREL error maps for the images presented in Fig. S1.

Figure S3. Intensity profiles across the region indicated in yellow for the nanowire samples presented in Fig. 2 of the main text.

Figure S4. SQUIRREL error maps for the images presented in Fig. 3 in the main text.

Figure. S5. Intensity profiles across regions indicated in yellow for the images presented in Fig. 3 in the main text.

Figure S6. Intensity profiles across the indicated line for the tendon image in Fig. 3 in the main text. The relative intensity of the indicated peaks is clearly not preserved in the super-resolution reconstruction.

Figure S7. SQUIRREL error maps for the images presented in Fig. 5 in the main text.

Table S1: Parameters used for SRRF processing of images presented in the main text.

| Figure | Magnification | Ring Radius (pixels) |
| --- | --- | --- |
| Figure 1 | 2 | 1.5 |
| Figure 2 (both images) | 3 | 1.6 |
| Figure 3 BaTi | 2 | 2.2 |
| Figure 3 Starch | 2 | 1.5 |
| Figure 3 Tendon | 1 | 1.6 |
| Figure 5 | 1 | 3 |

Table S2: Parameters used for MSSR processing of images presented in the main text.

| Figure | Magnification | MSSR Order |
| --- | --- | --- |
| Figure 1 | 2 | 2 |
| Figure 2 (both images) | 2 | 1 |
| Figure 3 BaTi | 2 | 0 |
| Figure 3 Starch | 2 | 1 |
| Figure 3 Tendon | 2 | 1 |
| All SRRF + MSSR images | 1 | 0 |

Table S3: Parameters used for DPR processing of images presented in the main text.

| Figure | Gain |
| --- | --- |
| Figure 1 | 2 |
| Figure 2 (both images) | 2 |
| Figure 3 BaTi | 2 |
| Figure 3 Starch | 2 |
| Figure 3 Tendon | 2 |
| Figure 5 | 1 |
